# Supplementary material for: Untargeted Metagenomic Investigation of the Airway Microbiome of Cystic Fibrosis Patients with Moderate-Severe Lung Disease
Source: Microorganisms. 2020 Jul 4;8(7):1003. doi: 10.3390/microorganisms8071003 (PMC7409339; doi:10.3390/microorganisms8071003)
Supplement: Supplementary file 1 [file microorganisms-08-01003-s001.zip › Supplementary /Figure_S8.pdf]

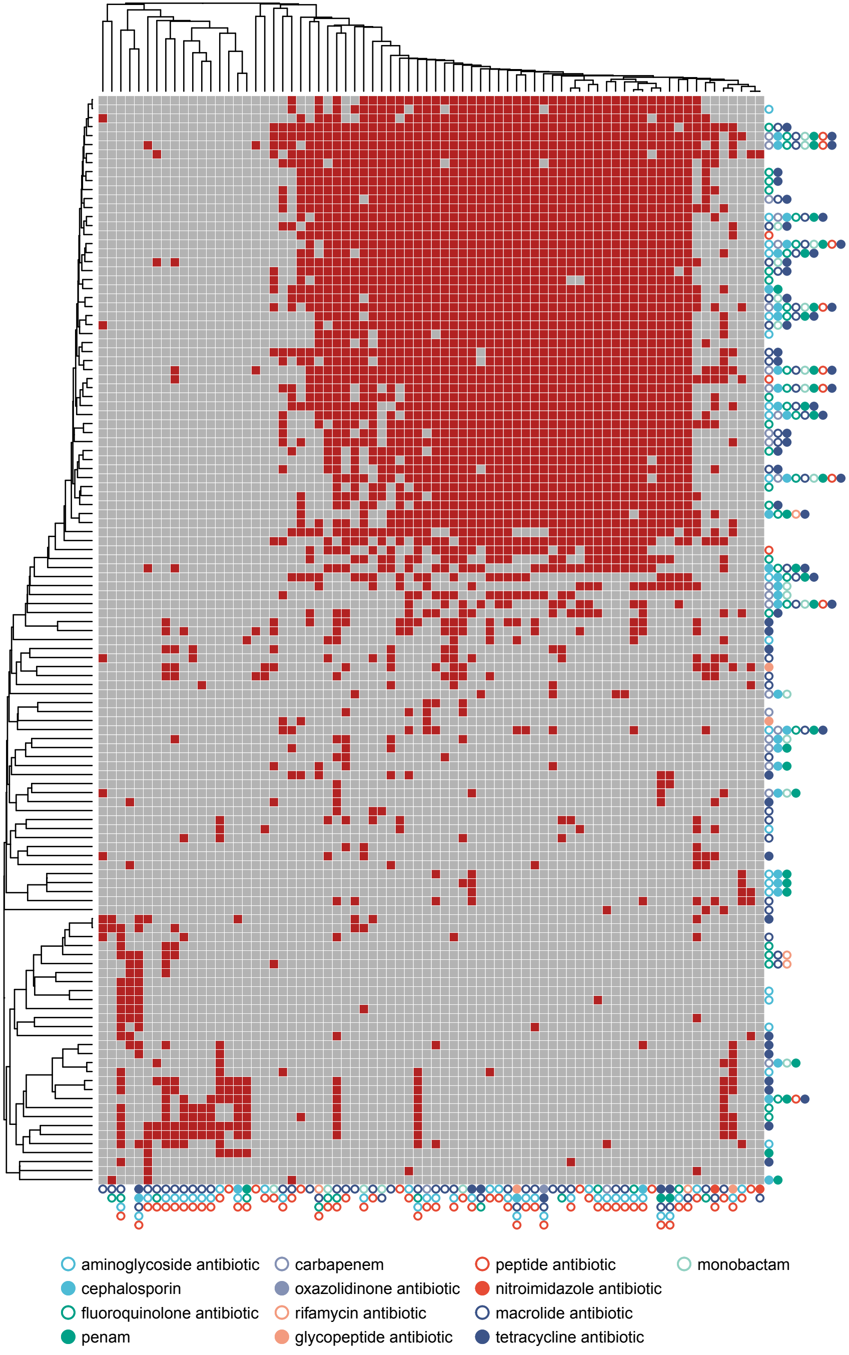
aminoglycoside antibiotic
cephalosporin
fluoroquinolone antibiotic
penam
carbapenem
oxazolidinone antibiotic
rifamycin antibiotic
glycopeptide antibiotic
peptide antibiotic
nitroimidazole antibiotic
macrolide antibiotic
tetracycline antibiotic
monobactam
