# Supplementary material for: Untargeted Metagenomic Investigation of the Airway Microbiome of Cystic Fibrosis Patients with Moderate-Severe Lung Disease
Source: Microorganisms. 2020 Jul 4;8(7):1003. doi: 10.3390/microorganisms8071003 (PMC7409339; doi:10.3390/microorganisms8071003)
Supplement: Supplementary file 1 [file microorganisms-08-01003-s001.zip › Supplementary /microorganisms_826830_Supplementary.docx]

Supplementary

Untargeted metagenomic investigation of the airway microbiome of cystic fibrosis patients with moderate-severe lung disease

Giovanni Bacci ^1^, Giovanni Taccetti ^2^, Daniela Dolce ^2^, Federica Armanini ^3^, Nicola Segata ^3^, Francesca Di Cesare ^1^, Vincenzina Lucidi ^4^, Ersilia Fiscarelli ^4^, Patrizia Morelli ^5^, Rosaria Casciaro ^5^, Anna Negroni ^6^, Alessio Mengoni ^1^ and Annamaria Bevivino ^6*^

^1^Department of Biology, University of Florence, Sesto Fiorentino, 50019 Florence, Italy; giovanni.bacci@unifi.it (G.B.), francesca.dicesare@stud.unifi.it (F.D.C.), alessio.mengoni@unifi.it (A.M.)

^2^Cystic Fibrosis Center, Anna Meyer Children’s University Hospital, Department of Pediatrics Medicine, 50139 Florence, Italy; giovanni.taccetti@meyer.it (G.T.), daniela.dolce@meyer.it (D.D.)

^3^Centre for Integrative Biology, University of Trento, 38122 Trento, Italy; federica.armanini@unitn.it (F.A.), nicola.segata@unitn.it (N.S.)

^4^Children’s Hospital and Research Institute Bambino Gesù, 00165 Rome, Italy; vincenzina.lucidi@opbg.net (V.L.), evita.fiscarelli@opbg.net (E.F.)

^5^Cystic Fibrosis Center, IRCCS G. Gaslini Institute, Department of Pediatrics, 16147 Genoa, Italy; PatriziaMorelli@gaslini.org (P.M.), rosariacasciaro@gaslini.org (R.C.)

^6^Department for Sustainability, Italian National Agency for New Technologies, Energy and Sustainable Economic Development, ENEA Casaccia Research Center, 00123 Rome, Italy; anna.negroni@enea.it (A.N.); annamaria.bevivino@enea.it (A.B.)

***** Correspondence: annamaria.bevivino@enea.it; Tel: +390630483868

**TABLE S1.** Samples and patients’ characteristics for each sample collected during the study.

**TABLE S2.** Number of reads for each sample.

| **ID** | **Time point** | **# reads** | **# pairs** | **# unpaired** |
| --- | --- | --- | --- | --- |
| B01 | 0 | 5503844 | 2325382 | 853080 |
| B01 | 1 | 868905 | 406838 | 55229 |
| B01 | 2 | 504486 | 214496 | 75494 |
| B01 | 3 | 1123511 | 530841 | 61829 |
| B01 | 1 | 1288912 | 623800 | 41312 |
| B02 | 0 | 1502243 | 623532 | 255179 |
| B02 | 2 | 8991858 | 4451619 | 88620 |
| B02 | 3 | 5143954 | 2488108 | 167738 |
| B03 | 0 | 658063 | 190398 | 277267 |
| B03 | 1 | 2115889 | 1025708 | 64473 |
| B03 | 2 | 10714542 | 5261449 | 191644 |
| B03 | 3 | 7484908 | 3638635 | 207638 |
| B06 | 0 | 5069581 | 2234180 | 601221 |
| B06 | 1 | 2070381 | 996544 | 77293 |
| B06 | 2 | 13180766 | 6517309 | 146148 |
| B06 | 3 | 24111405 | 11622361 | 866683 |
| G10 | 0 | 10932913 | 4877651 | 1177611 |
| G10 | 1 | 14514555 | 7134118 | 246319 |
| G10 | 2 | 3628618 | 1751868 | 124882 |
| G10 | 3 | 5452496 | 2625377 | 201742 |
| G24 | 0 | 785844 | 320254 | 145336 |
| G24 | 1 | 995004 | 435853 | 123298 |
| G24 | 2 | 3536639 | 1728879 | 78881 |
| G28 | 0 | 5760251 | 2518915 | 722421 |
| G28 | 1 | 661864 | 321914 | 18036 |
| G30 | 0 | 1836789 | 678840 | 479109 |
| G31 | 0 | 242296 | 102687 | 36922 |
| G31 | 1 | 1777716 | 859179 | 59358 |
| G34 | 1 | 605036 | 281046 | 42944 |
| M05 | 0 | 12125170 | 5264493 | 1596184 |
| M05 | 1 | 1124272 | 496383 | 131506 |
| M05 | 2 | 1470732 | 701131 | 68470 |
| M05 | 3 | 1153167 | 533168 | 86831 |
| M19 | 0 | 6259797 | 2704837 | 850123 |
| M19 | 1 | 17369379 | 8572310 | 224759 |
| M19 | 2 | 9391369 | 4639094 | 113181 |
| M19 | 3 | 9318334 | 4611450 | 95434 |
| M21 | 0 | 3128755 | 1369530 | 389695 |
| M21 | 1 | 224963 | 74970 | 75023 |
| M21 | 2 | 1335393 | 628615 | 78163 |
| M21 | 1 | 216831 | 58002 | 100827 |
| M22 | 0 | 3088535 | 1311593 | 465349 |
| M22 | 1 | 806490 | 381277 | 43936 |
| M22 | 2 | 1496116 | 721477 | 53162 |
| M22 | 3 | 819739 | 321497 | 176745 |
| M22 | 1 | 831267 | 394417 | 42433 |
| M23 | 0 | 964452 | 413639 | 137174 |
| M23 | 1 | 2031131 | 986181 | 58769 |
| M23 | 2 | 991655 | 466705 | 58245 |
| M23 | 1 | 970320 | 456575 | 57170 |
| M24 | 0 | 6030041 | 2542675 | 944691 |
| M24 | 1 | 922 | 446 | 30 |
| M24 | 2 | 31295587 | 15469995 | 355597 |
| M24 | 3 | 16800705 | 8254483 | 291739 |
| M25 | 0 | 18762529 | 8411793 | 1938943 |
| M25 | 1 | 5227170 | 2569092 | 88986 |
| M25 | 2 | 535041 | 181216 | 172609 |
| M25 | 3 | 7454111 | 3658164 | 137783 |
| M26 | 0 | 2258264 | 979675 | 298914 |
| M26 | 1 | 8457325 | 4176615 | 104095 |
| M26 | 2 | 5689324 | 2797112 | 95100 |
| M26 | 3 | 2919836 | 1413367 | 93102 |
| M26 | 1 | 33719580 | 16657112 | 405356 |
| M28 | 0 | 3458580 | 1524503 | 409574 |
| M28 | 1 | 5827736 | 2853068 | 121600 |
| M28 | 2 | 3070091 | 1440938 | 188215 |
| M28 | 3 | 1029948 | 475535 | 78878 |
| M29 | 0 | 1500423 | 599852 | 300719 |
| M29 | 1 | 936022 | 411010 | 114002 |
| M29 | 2 | 2076152 | 995563 | 85026 |
| M29 | 3 | 521984 | 183202 | 155580 |
| M31 | 0 | 6846501 | 2877219 | 1092063 |
| M31 | 1 | 1238914 | 552508 | 133898 |
| M31 | 2 | 1391941 | 664028 | 63885 |
| M33 | 0 | 1188194 | 434000 | 320194 |
| M33 | 1 | 3693755 | 1810992 | 71771 |
| M33 | 2 | 3108154 | 1492259 | 123636 |
| M33 | 3 | 2562448 | 1246795 | 68858 |
| M33 | 1 | 5604678 | 2758664 | 87350 |

ID, sample’s ID; Time point, time point at which sample was collected; # reads, total number of reads after preprocessing; # pairs, number of paired reads; # unpaired, number of unpaired reads.

**TABLE S3.** Summary of all species detected with a mean abundance higher than 0.2%.

| Kingdom | Phylum | Class | Order | Family | Genus | Species | (Mean abundance ± standard error) % |
| --- | --- | --- | --- | --- | --- | --- | --- |
| Viruses | - | - | Caudovirales | Siphoviridae | Lambdalikevirus | Enterobacteria_phage_lambda | 1.06 ± 0.508 |
|  |  |  |  | Podoviridae | Podoviridae_noname | Streptococcus_phage_Cp_1 | 0.30 ± 0.213 |
| Bacteria | Proteobacteria | Gammaproteobacteria | Pseudomonadales | Pseudomonadaceae | Pseudomonas | Pseudomonas_unclassified | 1.18 ± 0.494 |
|  |  |  |  |  |  | **Pseudomonas_aeruginosa** | 25.44 ± 3.657 |
|  |  |  | Pasteurellales | Pasteurellaceae | Haemophilus | Haemophilus_parainfluenzae | 0.95 ± 0.268 |
|  |  |  |  |  |  | Haemophilus_influenzae | 0.41 ± 0.331 |
|  |  | Betaproteobacteria | Neisseriales | Neisseriaceae | Neisseria | Neisseria_unclassified | 0.69 ± 0.308 |
|  |  |  |  |  |  | Neisseria_flavescens | 0.52 ± 0.226 |
|  |  |  | Burkholderiales | Burkholderiaceae | Ralstonia | Ralstonia_unclassified | 0.55 ± 0.258 |
|  |  |  |  |  | Lautropia | Lautropia_mirabilis | 0.22 ± 0.167 |
|  | Fusobacteria | Fusobacteriia | Fusobacteriales | Fusobacteriaceae | Fusobacterium | Fusobacterium_nucleatum | 0.30 ± 0.069 |
|  | Firmicutes | Negativicutes | Selenomonadales | Veillonellaceae | Veillonella | **Veillonella_unclassified** | 2.77 ± 0.470 |
|  |  |  |  |  |  | Veillonella_parvula | 1.76 ± 0.434 |
|  |  |  |  |  |  | Veillonella_dispar | 0.34 ± 0.093 |
|  |  |  |  |  |  | Veillonella_atypica | 0.66 ± 0.155 |
|  |  | Erysipelotrichia | Erysipelotrichales | Erysipelotrichaceae | Solobacterium | Solobacterium_moorei | 0.41 ± 0.118 |
|  |  | Bacilli | Lactobacillales | Streptococcaceae | Streptococcus | Streptococcus_salivarius | 0.45 ± 0.188 |
|  |  |  |  |  |  | **Streptococcus_parasanguinis** | 7.02 ± 1.091 |
|  |  |  |  |  |  | Streptococcus_mitis_oralis_pneumoniae | 1.19 ± 0.217 |
|  |  |  |  |  |  | Streptococcus_infantis | 1.11 ± 0.266 |
|  |  |  |  |  |  | Streptococcus_australis | 0.65 ± 0.196 |
|  |  |  | Lactobacillales | Enterococcaceae | Enterococcus | **Enterococcus_faecalis** | 2.45 ± 1.421 |
|  |  |  |  | Carnobacteriaceae | Granulicatella | Granulicatella_unclassified | 1.87 ± 0.269 |
|  |  |  |  |  |  | Granulicatella_adiacens | 1.19 ± 0.157 |
|  |  |  |  | Aerococcaceae | Abiotrophia | Abiotrophia_defectiva | 0.40 ± 0.187 |
|  |  |  | Bacillales | Staphylococcaceae | Staphylococcus | **Staphylococcus_aureus** | 11.19 ± 2.833 |
|  |  |  |  | Bacillales_noname | Gemella | **Gemella_sanguinis** | 1.89 ± 0.386 |
|  |  |  |  |  |  | Gemella_morbillorum | 0.51 ± 0.203 |
|  |  |  |  |  |  | Gemella_haemolysans | 1.39 ± 0.373 |
|  | Bacteroidetes | Flavobacteriia | Flavobacteriales | Flavobacteriaceae | Capnocytophaga | Capnocytophaga_unclassified | 0.41 ± 0.097 |
|  |  |  |  |  |  | Capnocytophaga_sp_oral_taxon_329 | 0.34 ± 0.214 |
|  |  |  |  |  |  | Capnocytophaga_gingivalis | 0.37 ± 0.105 |
|  |  | Bacteroidia | Bacteroidales | Prevotellaceae | Prevotella | Prevotella_sp_C561 | 0.23 ± 0.153 |
|  |  |  |  |  |  | Prevotella_pleuritidis | 0.62 ± 0.336 |
|  |  |  |  |  |  | Prevotella_pallens | 0.21 ± 0.056 |
|  |  |  |  |  |  | Prevotella_nanceiensis | 0.51 ± 0.207 |
|  |  |  |  |  |  | **Prevotella_melaninogenica** | 2.89 ± 0.733 |
|  |  |  |  |  |  | Prevotella_histicola | 0.77 ± 0.397 |
|  |  |  |  | Porphyromonadaceae | Porphyromonas | **Porphyromonas_sp_oral_taxon_279** | 4.03 ± 0.947 |
|  | Actinobacteria | Actinobacteria | Coriobacteriales | Coriobacteriaceae | Atopobium | Atopobium_parvulum | 0.21 ± 0.047 |
|  |  |  | Bifidobacteriales | Bifidobacteriaceae | Bifidobacterium | Bifidobacterium_longum | 0.38 ± 0.351 |
|  |  |  | Actinomycetales | Micrococcaceae | Rothia | **Rothia_mucilaginosa** | 9.07 ± 1.378 |
|  |  |  |  |  |  | **Rothia_dentocariosa** | 3.00 ± 0.568 |
|  |  |  |  |  |  | Rothia_aeria | 0.54 ± 0.246 |
|  |  |  |  | Actinomycetaceae | Actinomyces | Actinomyces_graevenitzii | 1.53 ± 0.478 |

Top 10 species detected were reported in bold.

**TABLE S4.** Analysis of variance on alpha diversity indices.

|  | **Df** | **Sum Sq** | **Mean Sq** | **F value** | **Pr(>F)** |
| --- | --- | --- | --- | --- | --- |
| **Taxonomic profiling** | | | | | |
| ***Shannon index*** |  |  |  |  |  |
| Status | **2** | **3.57** | **1.78** | **3.73** | **0.0310** |
| Genotype | **1** | **2.34** | **2.34** | **4.89** | **0.0317** |
| Sample | **18** | **27.48** | **1.53** | **3.20** | **0.0006** |
| **FEV_1_ value** | 1 | 1.01 | 1.01 | 2.12 | 0.1521 |
| **Days** | 1 | 0.06 | 0.06 | 0.13 | 0.7208 |
| **Status:Genotype** | 1 | 1.69 | 1.69 | 3.55 | 0.0656 |
| **Residuals** | 49 | 23.41 | 0.48 |  |  |
|  |  |  |  |  |  |
| ***Inverse Simpson index*** |  |  |  |  |  |
| Status | **2** | **54.14** | **27.07** | **3.27** | **0.0466** |
| Genotype | **1** | **64.24** | **64.24** | **7.75** | **0.0076** |
| Sample | **18** | **554.16** | **30.79** | **3.71** | **0.0001** |
| **FEV_1_ value** | 1 | 20.81 | 20.81 | 2.51 | 0.1195 |
| **Days** | 1 | 0.17 | 0.17 | 0.02 | 0.8878 |
| **Status:Genotype** | 1 | 7.56 | 7.56 | 0.91 | 0.3442 |
| **Residuals** | 49 | 406.11 | 8.29 |  |  |
|  |  |  |  |  |  |
| **Pathway distribution** | | | | | |
| ***Shannon index*** |  |  |  |  |  |
| **Status** | 2 | 0.45 | 0.22 | 3.12 | 0.0533 |
| **Genotype** | 1 | 0.11 | 0.11 | 1.55 | 0.2189 |
| Sample | **18** | **3.90** | **0.22** | **3.03** | **0.0011** |
| **FEV_1_ value** | 1 | 0.19 | 0.19 | 2.62 | 0.1119 |
| **Days** | 1 | 0.08 | 0.08 | 1.06 | 0.3086 |
| **Status:Genotype** | 1 | 0.27 | 0.27 | 3.75 | 0.0587 |
| **Residuals** | 49 | 3.50 | 0.07 |  |  |
|  |  |  |  |  |  |
| ***Inverse Simpson index*** |  |  |  |  |  |
| Status | **2** | **4056.46** | **2028.23** | **3.57** | **0.0358** |
| **Genotype** | 1 | 269.70 | 269.70 | 0.47 | 0.4942 |
| Sample | **18** | **32244.56** | **1791.36** | **3.15** | **0.0007** |
| **FEV_1_ value** | 1 | 2010.30 | 2010.30 | 3.54 | 0.0660 |
| **Days** | 1 | 225.89 | 225.89 | 0.40 | 0.5314 |
| **Status:Genotype** | 1 | 2248.92 | 2248.92 | 3.96 | 0.0523 |
| **Residuals** | 49 | 27852.86 | 568.43 |  |  |

Results of analysis of variance (ANOVA) on Shannon and Inverse Simpson indices were reported in table. Results obtained from distribution of taxa and pathways were reported. Factors with a p-value lower than 0.05 were reported in bold.

**TABLE S5.** Tukey post hoc test on alpha diversity indices.

|  | Index | Contrast | Mean diff. | 2.5% (C.I.) | 97.5% (C.I.) | p-value |
| --- | --- | --- | --- | --- | --- | --- |
| Taxonomic profiling | | | | | | |
| Genotype |  |  |  |  |  |  |
|  | Shannon | HO – HE | 0.35 | 0.02 | 0.69 | 0.0405* |
|  | InvSimpson | HO – HE | 1.84 | 0.45 | 3.24 | 0.0108* |
| Status |  |  |  |  |  |  |
|  | Shannon | BL - TR | 0.67 | 0.04 | 1.30 | 0.0363* |
|  |  | RC - TR | 0.35 | -0.47 | 1.16 | 0.5631 |
|  |  | RC – BL | -0.32 | -0.92 | 0.28 | 0.4048 |
|  | InvSimpson | BL - TR | 2.52 | -0.11 | 5.15 | 0.0328* |
|  |  | RC - TR | 1.10 | -2.28 | 4.49 | 0.7113 |
|  |  | RC – BL | -1.41 | -3.91 | 1.08 | 0.3646 |
| Pathway distribution | | | | | | |
| Genotype |  |  |  |  |  |  |
|  | Shannon | HO – HE | 0.08 | -0.05 | 0.21 | 0.2416 |
|  | InvSimpson | HO – HE | 3.77 | -7.80 | 15.34 | 0.5151 |
| Status |  |  |  |  |  |  |
|  | Shannon | BL - TR | 0.22 | -0.02 | 0.47 | 0.0813 |
|  |  | RC - TR | 0.08 | -0.23 | 0.40 | 0.8017 |
|  |  | RC – BL | -0.14 | -0.37 | 0.09 | 0.3212 |
|  | InvSimpson | BL - TR | 21.45 | -0.30 | 43.21 | 0.0540 |
|  |  | RC - TR | 8.57 | -19.43 | 36.57 | 0.7409 |
|  |  | RC – BL | -12.88 | -33.54 | 7.79 | 0.2971 |

Factors were reported in bold whereas alpha diversity indices were reported once for all contrasts performed. Contrast were reported using the following abbreviations: HO, homozygote ΔF_508_; HE, heterozygote ΔF_508_; BL, baseline; TR, treatment (samples collected during the treatment of an exacerbation event); RC, recovery (the first sample collected after the end of an exacerbation event). The differences between the mean values of the given contrast were reported in the Mean diff. columns whereas the confidence interval was reported using the C.I. abbreviation. Contrasts reporting a p-value lower than 0.05 were marked with an asterisk.

**TABLE S6.** List of metabolic pathways identified in assembled reads. The Metacyc ID of the pathway was reported in column one whereas the complete path description was reported in column two.

**TABLE S7.** Tukey post hoc test on Sorensen similarity index.

|  | Contrast | Mean diff. | 2.5% (C.I.) | 97.5% (C.I.) | p-value |
| --- | --- | --- | --- | --- | --- |
| Genotype |  |  |  |  |  |
|  | Taxa - Pathways | 0.56 | 0.53 | 0.58 | < 0.0001* |
|  | HO - HE_t_ | 0.00 | -0.03 | 0.03 | 0.9805 |
| Status |  |  |  |  |  |
|  | Taxa - Pathways | 0.56 | 0.54 | 0.58 | < 0.0001* |
|  | BL - TR | -0.03 | -0.07 | 0.01 | 0.2704 |
|  | RC - TR | 0.01 | -0.05 | 0.06 | 0.9662 |
|  | RC – BL | 0.03 | -0.01 | 0.07 | 0.1027 |

Grouping factors were reported in bold whereas contrasts were reported using the following abbreviations: HO, homozygote ΔF_508_; HE, heterozygote ΔF_508_; BL, baseline; TR, treatment (samples collected during the treatment of an exacerbation event); RC, recovery (the first sample collected after the end of an exacerbation event). The differences between the mean values of the given contrast were reported in the Mean diff. column whereas the confidence interval was reported using the C.I. abbreviation. Contrasts with a pvalue lower than 0.05 were marked with an asterisk.

**TABLE S8.** Metabolic pathways differentially distributed across treatment statuses.

| Genotype | Contrast | MetaCyc name | Path description | logFC | AveExpr | t | P.Value | adj.P.Val |
| --- | --- | --- | --- | --- | --- | --- | --- | --- |
| Homozygote | TR Vs BL | PWY0-162 | superpathway of pyrimidine ribonucleotides de novo biosynthesis | -6.51 | 7.31 | -4.86 | 0.0000 | 0.0018 |
| Homozygote | TR Vs BL | PWY-6147 | 6-hydroxymethyl-dihydropterin diphosphate biosynthesis I | -6.71 | 7.17 | -4.45 | 0.0000 | 0.0043 |
| Homozygote | TR Vs BL | PWY-7539 | 6-hydroxymethyl-dihydropterin diphosphate biosynthesis III (Chlamydia) | -6.13 | 6.71 | -4.32 | 0.0000 | 0.0046 |
| Homozygote | TR Vs BL | PWY-7199 | pyrimidine deoxyribonucleosides salvage | -6.33 | 7.14 | -4.15 | 0.0001 | 0.0065 |
| Homozygote | TR Vs BL | PWY-6628 | superpathway of L-phenylalanine biosynthesis | -6.01 | 6.86 | -3.91 | 0.0002 | 0.0123 |
| Homozygote | TR Vs BL | DAPLYSINESYN-PWY | L-lysine biosynthesis I | -5.75 | 6.13 | -3.81 | 0.0003 | 0.0123 |
| Homozygote | TR Vs BL | PWY-6703 | preQ0 biosynthesis | -5.83 | 6.28 | -3.79 | 0.0003 | 0.0123 |
| Homozygote | TR Vs BL | PENTOSE-P-PWY | pentose phosphate pathway | -5.51 | 6.28 | -3.77 | 0.0003 | 0.0123 |
| Homozygote | TR Vs BL | PWY0-1319 | CDP-diacylglycerol biosynthesis II | -6.29 | 6.49 | -3.70 | 0.0004 | 0.0142 |
| Homozygote | TR Vs BL | PWY-7187 | pyrimidine deoxyribonucleotides de novo biosynthesis II | -5.78 | 6.48 | -3.54 | 0.0006 | 0.0191 |
| Homozygote | TR Vs BL | PWY-621 | sucrose degradation III (sucrose invertase) | -5.56 | 6.12 | -3.53 | 0.0007 | 0.0191 |
| Homozygote | TR Vs BL | DENOVOPURINE2-PWY | superpathway of purine nucleotides de novo biosynthesis II | -5.89 | 6.59 | -3.52 | 0.0007 | 0.0191 |
| Homozygote | TR Vs BL | PWY-5667 | CDP-diacylglycerol biosynthesis I | -5.91 | 6.24 | -3.46 | 0.0009 | 0.0216 |
| Homozygote | TR Vs BL | P4-PWY | superpathway of L-lysine, L-threonine and L-methionine biosynthesis I | -5.62 | 5.80 | -3.30 | 0.0014 | 0.0299 |
| Homozygote | TR Vs BL | PWY66-409 | superpathway of purine nucleotide salvage | -5.84 | 6.38 | -3.27 | 0.0016 | 0.0299 |
| Homozygote | TR Vs BL | MET-SAM-PWY | superpathway of S-adenosyl-L-methionine biosynthesis | -5.05 | 7.00 | -3.23 | 0.0018 | 0.0299 |
| Homozygote | TR Vs BL | RIBOSYN2-PWY | flavin biosynthesis I (bacteria and plants) | -5.03 | 5.73 | -3.15 | 0.0023 | 0.0319 |
| Homozygote | RC Vs BL | PWY-5188 | tetrapyrrole biosynthesis I (from glutamate) | -6.95 | 8.24 | -8.14 | 0.0000 | 0.0000 |
| Homozygote | RC Vs BL | HEMESYN2-PWY | heme b biosynthesis II (anaerobic) | -6.63 | 7.82 | -5.65 | 0.0000 | 0.0000 |
| Homozygote | RC Vs BL | ARGSYN-PWY | L-arginine biosynthesis I (via L-ornithine) | -6.33 | 7.59 | -5.42 | 0.0000 | 0.0001 |
| Homozygote | RC Vs BL | PWY-7400 | L-arginine biosynthesis IV (archaebacteria) | -6.21 | 7.54 | -5.12 | 0.0000 | 0.0002 |
| Homozygote | RC Vs BL | GLUTORN-PWY | L-ornithine biosynthesis I | -5.53 | 7.02 | -4.53 | 0.0000 | 0.0009 |
| Homozygote | RC Vs BL | PWY-5918 | superpathay of heme b biosynthesis from glutamate | -5.97 | 7.36 | -4.40 | 0.0000 | 0.0012 |
| Homozygote | RC Vs BL | RHAMCAT-PWY | L-rhamnose degradation I | 6.73 | 3.12 | 4.38 | 0.0000 | 0.0012 |
| Homozygote | RC Vs BL | HEME-BIOSYNTHESIS-II | heme b biosynthesis I (aerobic) | -5.53 | 7.09 | -4.16 | 0.0001 | 0.0023 |
| Homozygote | RC Vs BL | PWY-5189 | tetrapyrrole biosynthesis II (from glycine) | -5.05 | 7.33 | -3.95 | 0.0002 | 0.0044 |
| Homozygote | RC Vs BL | PWY0-162 | superpathway of pyrimidine ribonucleotides de novo biosynthesis | -6.47 | 7.31 | -3.45 | 0.0009 | 0.0161 |
| Homozygote | RC Vs BL | PWY-6147 | 6-hydroxymethyl-dihydropterin diphosphate biosynthesis I | -6.74 | 7.17 | -3.20 | 0.0019 | 0.0275 |
| Homozygote | RC Vs BL | PWY-6897 | thiamine salvage II | -6.28 | 6.54 | -3.10 | 0.0026 | 0.0355 |
| Homozygote | RC Vs BL | PWY-7539 | 6-hydroxymethyl-dihydropterin diphosphate biosynthesis III (Chlamydia) | -6.11 | 6.71 | -3.08 | 0.0028 | 0.0367 |
| Homozygote | RC Vs TR | PWY-5188 | tetrapyrrole biosynthesis I (from glutamate) | -6.84 | 8.24 | -7.17 | 0.0000 | 0.0000 |
| Homozygote | RC Vs TR | ARGSYN-PWY | L-arginine biosynthesis I (via L-ornithine) | -6.75 | 7.59 | -5.17 | 0.0000 | 0.0002 |
| Homozygote | RC Vs TR | RHAMCAT-PWY | L-rhamnose degradation I | 8.85 | 3.12 | 5.16 | 0.0000 | 0.0002 |
| Homozygote | RC Vs TR | PWY-7400 | L-arginine biosynthesis IV (archaebacteria) | -6.74 | 7.54 | -4.98 | 0.0000 | 0.0003 |
| Homozygote | RC Vs TR | HEMESYN2-PWY | heme b biosynthesis II (anaerobic) | -6.28 | 7.82 | -4.79 | 0.0000 | 0.0005 |
| Homozygote | RC Vs TR | GLUTORN-PWY | L-ornithine biosynthesis I | -5.94 | 7.02 | -4.36 | 0.0000 | 0.0020 |
| Homozygote | RC Vs TR | PWY-5189 | tetrapyrrole biosynthesis II (from glycine) | -5.47 | 7.33 | -3.83 | 0.0002 | 0.0090 |
| Homozygote | RC Vs TR | PWY-5918 | superpathay of heme b biosynthesis from glutamate | -5.74 | 7.36 | -3.79 | 0.0003 | 0.0094 |
| Homozygote | RC Vs TR | PWY-7199 | pyrimidine deoxyribonucleosides salvage | 8.52 | 7.14 | 3.57 | 0.0006 | 0.0162 |
| Homozygote | RC Vs TR | HEME-BIOSYNTHESIS-II | heme b biosynthesis I (aerobic) | -5.20 | 7.09 | -3.50 | 0.0008 | 0.0188 |

Results of limma analysis were reported in the table together with the genotype, the pathway name (according to MetaCyc), and the description of the pathway. Other columns are: logFC, the log2-transformed fold change value; AveExpr, the average log2-expression level for that pathway across all samples; t, the t-value according to limma’s moderated t-test; P.Value, the p-value; adj.P.Val, the adjusted p-value using the Benjamini–Hochberg correction. Contrast were reported using the following abbreviations: Homozygote, homozygote ΔF_508_; BL, baseline; TR, treatment (samples collected during the treatment of an exacerbation event); RC, recovery (the first sample collected after the end of an exacerbation event). Only contrasts with an adjusted p-value lower than 0.05 and an absolute log fold-change value higher than 5 were reported.

**TABLE S9.** Antibiotic resistance genes differentially distributed across treatment statuses.

| Genotype | Contrast | Gene name | AR family | logFC | AveExpr | t | P.Value | adj.P.Val |
| --- | --- | --- | --- | --- | --- | --- | --- | --- |
| Heterozygote | RC Vs BL | PC1 beta-lactamase (blaZ) | blaZ beta-lactamase | 7.68 | 7.58 | 7.08 | 0.0000 | 0.0000 |
| Heterozygote | RC Vs BL | LRA-13 | class C LRA beta-lactamase; class D LRA beta-lactamase | 5.43 | 7.27 | 6.29 | 0.0000 | 0.0000 |
| Heterozygote | RC Vs TR | LRA-13 | class C LRA beta-lactamase; class D LRA beta-lactamase | 5.50 | 7.27 | 5.41 | 0.0000 | 0.0001 |
| Heterozygote | RC Vs TR | PC1 beta-lactamase (blaZ) | blaZ beta-lactamase | 7.91 | 7.58 | 5.26 | 0.0000 | 0.0001 |
| Heterozygote | RC Vs TR | sav1866 | ATP-binding cassette (ABC) antibiotic efflux pump | 6.50 | 8.78 | 3.89 | 0.0002 | 0.0050 |
| Heterozygote | RC Vs TR | tet(38) | major facilitator superfamily (MFS) antibiotic efflux pump | 6.95 | 9.00 | 3.60 | 0.0006 | 0.0118 |

Results of limma analysis were reported in the table together with the genotype, the antibiotic resistance gene name (according to the CARD database), and the antibiotic resistance family (AR family). Other columns are: logFC, the log2-transformed fold change value; AveExpr, the average log2-expression level for that pathway across all samples; t, the t-value according to limma’s moderated t-test; P.Value, the p-value; adj.P.Val, the adjusted pvalue using the Benjamini–Hochberg correction. Contrasts were reported using the following abbreviations: Homozygote, homozygote ΔF_508_; BL, baseline; TR, treatment (samples collected during the treatment of an exacerbation event); RC, recovery (the first sample collected after the end of an exacerbation event). Only contrasts with an adjusted p-value lower than 0.05 and an absolute log fold-change value higher than 5 were reported.

**Supplementary Figures**

**FIGURE S1.** Ordination analyses based on a) taxonomic assignments and b) pathway distribution detected with MetaPhlAn2 and HUMAnN2, respectively. Ordination analyses were conducted using the Bray-Curtis dissimilarity index and ordered following the principle coordinate decomposition method (PCoA). The percentage of variance explained by each coordinate was reported between round brackets. Homozygote and heterozygote refer to ΔF508 mutation of CFTR gene. BL, baseline; TR, treatment; RC, recovery.

**FIGURE S2.** Strain-level phylogenetic trees of all detected microbes in the study. Phylogenetic trees obtained through StrainPhlAn pipeline were reported for the main pathogenic signatures of CF disease. Only species with a set of known markers were included in the plot (as reported in the StrainPhlAn pipeline).

**FIGURE S3.** The effect of an exacerbation event (aka different antibiotic treatment) on alpha diversity inspected using both the Shannon index and the inverse Simpson index. Diversity indexes were computed for both a) taxonomic signature and b) metabolic pathways. BL, baseline; TR, treatment; RC, recovery. Each box shows the “interquartile range” (IQR) that is the differences between the third and the first quartile of data (the 75^th^ and the 25^th^ percentile). Horizontal bars are medians whereas whiskers represent the minimum and maximum values defined as Q1 – (1.5 x IQR) and Q3 + (1.5 x IQR), respectively. Observations that fell outside minimum and maximum values were defined as outliers and reported using white points.

**FIGURE S4.** Effect of genotypes and samples on the bacterial diversity of lung microbiome. The effect of a) genotype on alpha diversity was inspected together with b) the interindividual effect. Both the Shannon index and the inverse Simpson index were included in the analysis and reported in different panel. Contrasts reporting a p-value lower than 0.05 were reported using a single asterisk whereas those with a p-value lower than 0.01 were reported using two asterisks. Homozygote and heterozygote refer to ΔF_508_ mutation of CFTR gene. BL, baseline; TR, treatment (samples collected during the treatment of an exacerbation event); RC, recovery (the first sample collected after the end of an exacerbation event).

**FIGURE S5.** Volcano plot reporting results obtained with limma moderated t-test on pathway distribution. Differential abundant pathways were assessed through the limma moderated t-test and results were reported in this plot. Each panel report a different contrast between samples collected during the treatment of an exacerbation event (TR), after the resolution of an exacerbation event (RC), and during normal visits (BL). Contrasts were divided and grouped according to different genotypes and refers to the ΔF_508_ mutation of CFTR gene. Pathways reporting a significant difference (p-value < 0.05 and |log fold-change| > 5) in the contrast considered were reported in red otherwise were reported in blue. Gray points are those from other contrasts and were reported on the back of the plot.

**FIGURE S6.** Volcano plot reporting results obtained with limma moderated t-test on resistance gene distribution. Differential abundant ARGs were assessed through the limma moderated t-test and results were reported in this plot. For additional information about the plot see legend of Figure S5.

**FIGURE S7.** Effect of the antibiotic intake on the distribution of antibiotic resistance genes. Volcano plot reporting results obtained with limma moderated t-test for each class of antibiotic are shown. Differential abundant tests were performed for each class of antibiotic used in the study. For additional information about the plot see legend of Figure S3.

**FIGURE S8.** Antibiotic resistance map of each sample included in the study. Antibiotic resistance genes were reported in the y-axis whereas samples were reported in the x-axis. Antibiotic classes (both for ARG and for patient treatments) where reported using dots at the end of the heatmap. Hierarchical clustering was computed using the Jaccard index for binary data with the UPGMA method. Red cells correspond to the presence of a gene in samples whereas gray cell correspond to absence.
