# Supplementary material for: Untargeted Metagenomic Investigation of the Airway Microbiome of Cystic Fibrosis Patients with Moderate-Severe Lung Disease
Source: Microorganisms. 2020 Jul 4;8(7):1003. doi: 10.3390/microorganisms8071003 (PMC7409339; doi:10.3390/microorganisms8071003)
Supplement: Supplementary file 1 [file microorganisms-08-01003-s001.zip › Supplementary /Figure_S6.pdf]

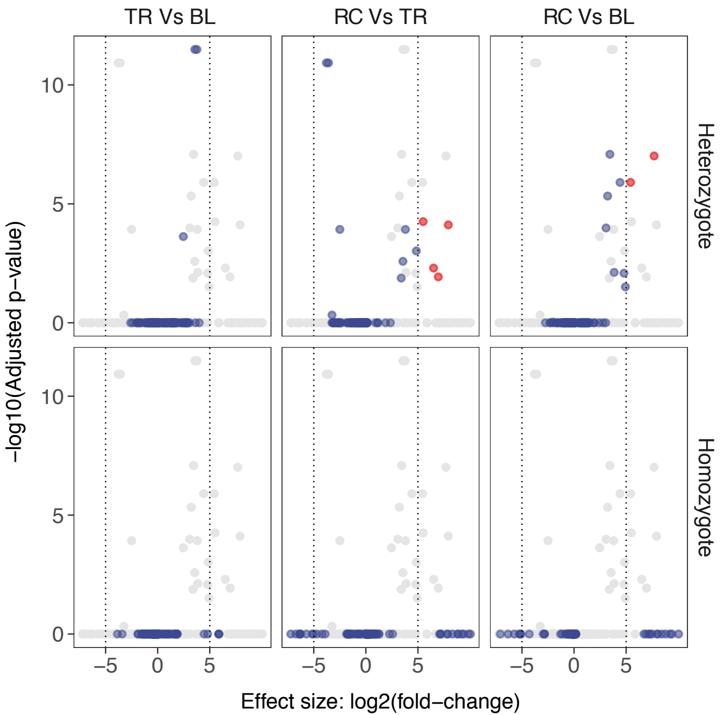
TR Vs BL
RC Vs TR
RC Vs BL
Heterozygote
Homozygote
−log10(Adjusted p−value)
Effect size: log2(fold−change)
10
5
0
−5
0
5
