# Supplementary material for: Untargeted Metagenomic Investigation of the Airway Microbiome of Cystic Fibrosis Patients with Moderate-Severe Lung Disease
Source: Microorganisms. 2020 Jul 4;8(7):1003. doi: 10.3390/microorganisms8071003 (PMC7409339; doi:10.3390/microorganisms8071003)
Supplement: Supplementary file 1 [file microorganisms-08-01003-s001.zip › Supplementary /Figure_S7.pdf]

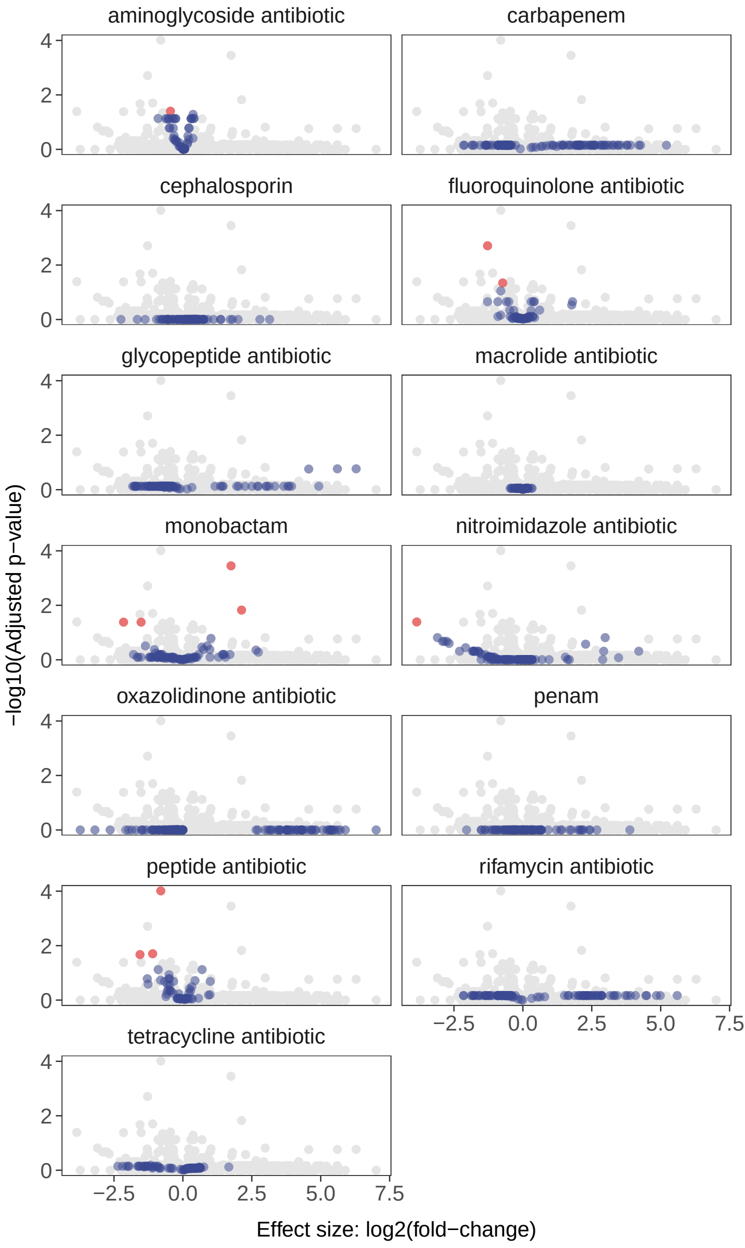

aminoglycoside antibiotic
carbapenem
cephalosporin
fluoroquinolone antibiotic
glycopeptide antibiotic
macrolide antibiotic
monobactam
nitroimidazole antibiotic
oxazolidinone antibiotic
penam
peptide antibiotic
rifamycin antibiotic
tetracycline antibiotic
−log10(Adjusted p−value)
0
2
4
−2.5
0.0
2.5
5.0
7.5
Effect size: log2(fold−change)
