# Supplementary figures and images for: Untargeted Metagenomic Investigation of the Airway Microbiome of Cystic Fibrosis Patients with Moderate-Severe Lung Disease
Source: Microorganisms. 2020 Jul 4;8(7):1003. doi: 10.3390/microorganisms8071003 (PMC7409339; doi:10.3390/microorganisms8071003)

**a**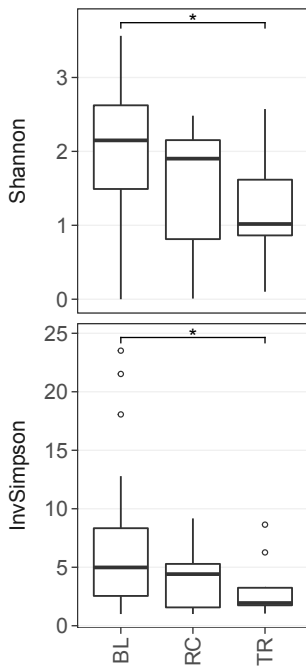**b**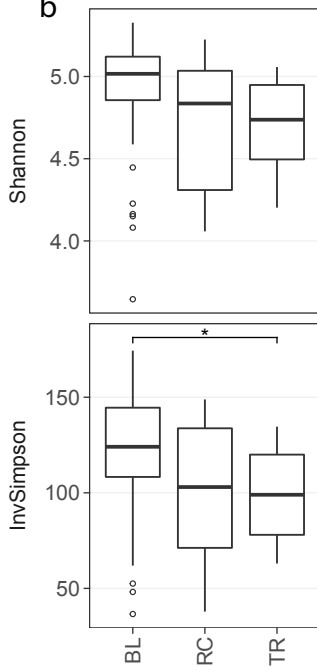

Supplement: Supplementary file 1 [file microorganisms-08-01003-s001.zip › Supplementary /Figure_S3.pdf]

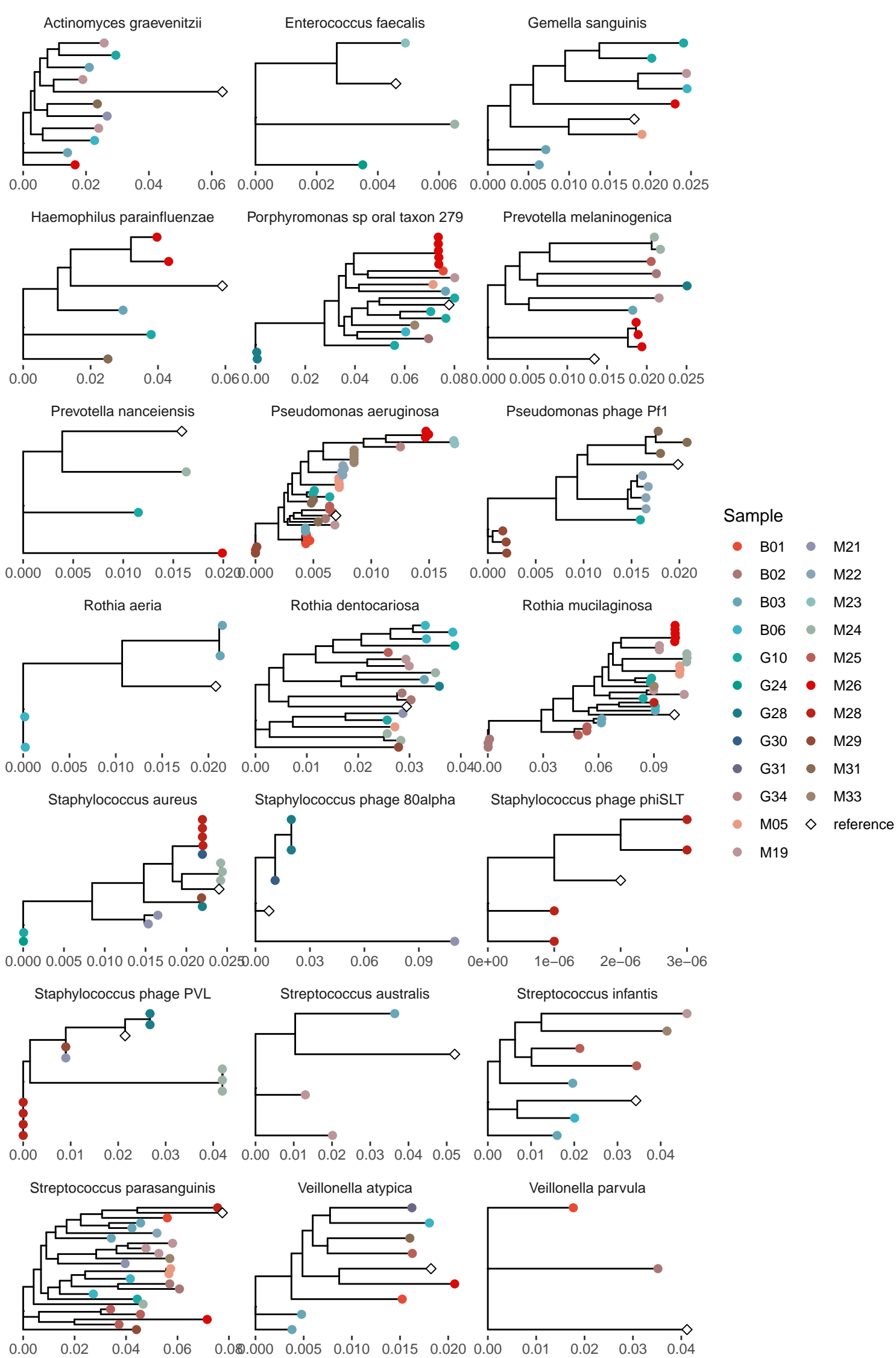

Supplement: Supplementary file 1 [file microorganisms-08-01003-s001.zip › Supplementary /Figure_S2.pdf]

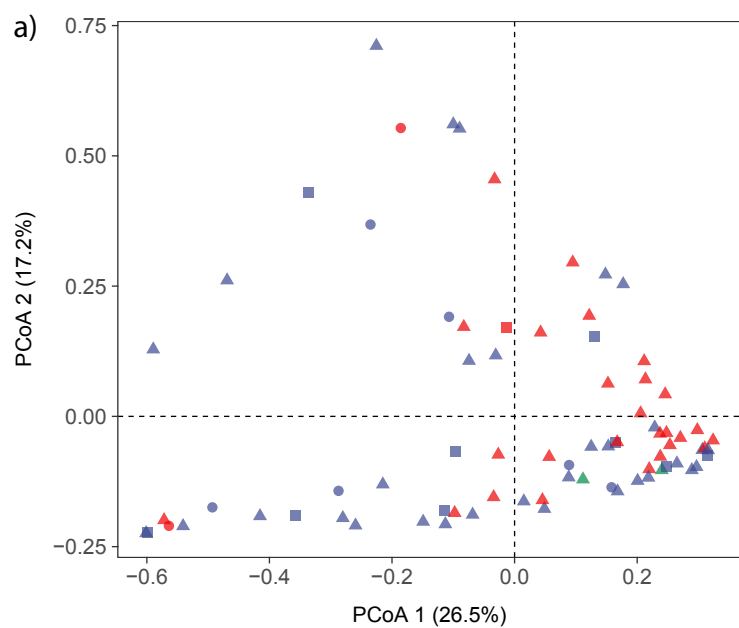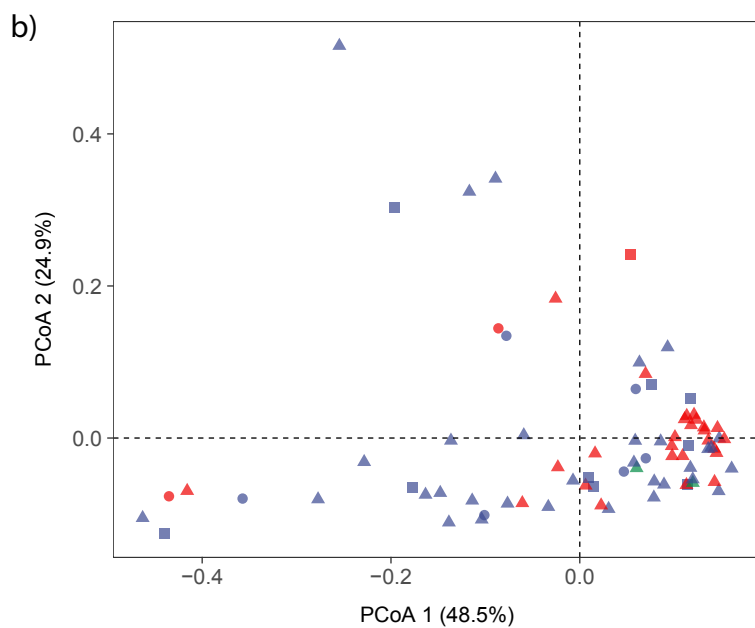

**Genotype** ● heterozygote ● homozygote ● other

**Status** ● TR ▲ BL ■ RC

Supplement: Supplementary file 1 [file microorganisms-08-01003-s001.zip › Supplementary /Figure_S1.pdf]

TR Vs BL

RC Vs TR

RC Vs BL

Heterozygote

Homozygote

 $-\log_{10}(\text{Adjusted p-value})$ 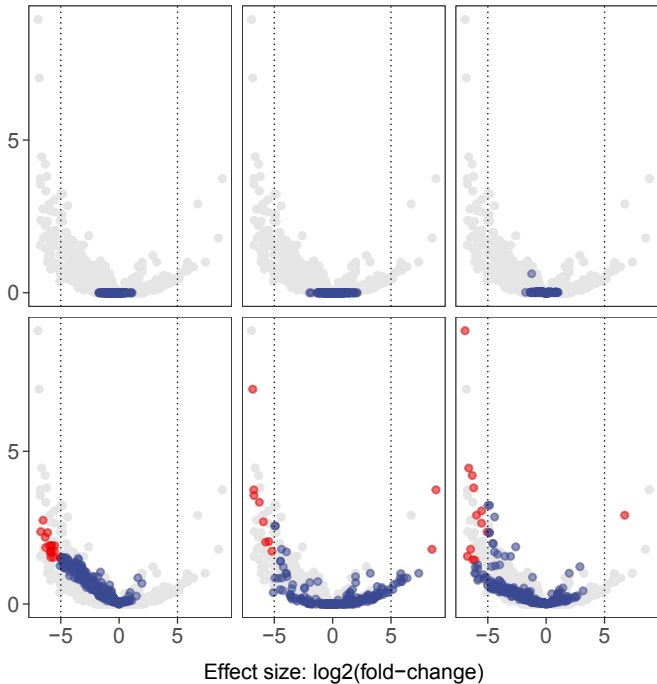

Supplement: Supplementary file 1 [file microorganisms-08-01003-s001.zip › Supplementary /Figure_S5.pdf]

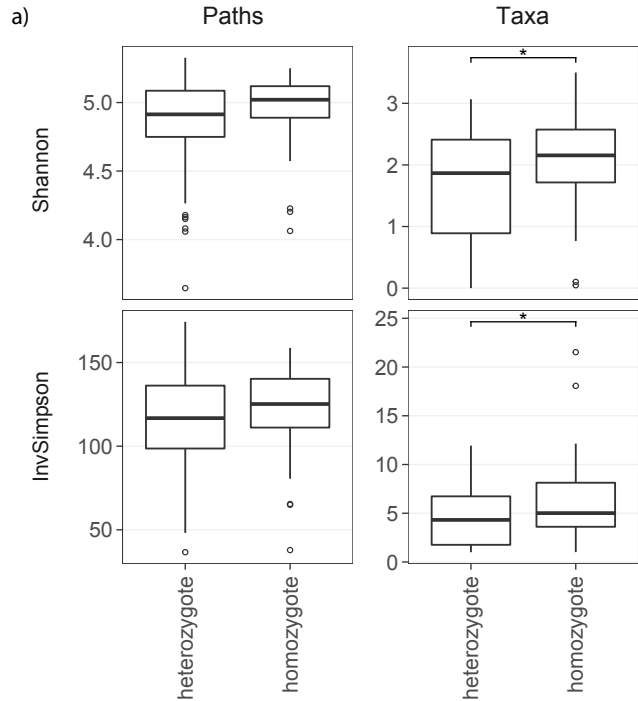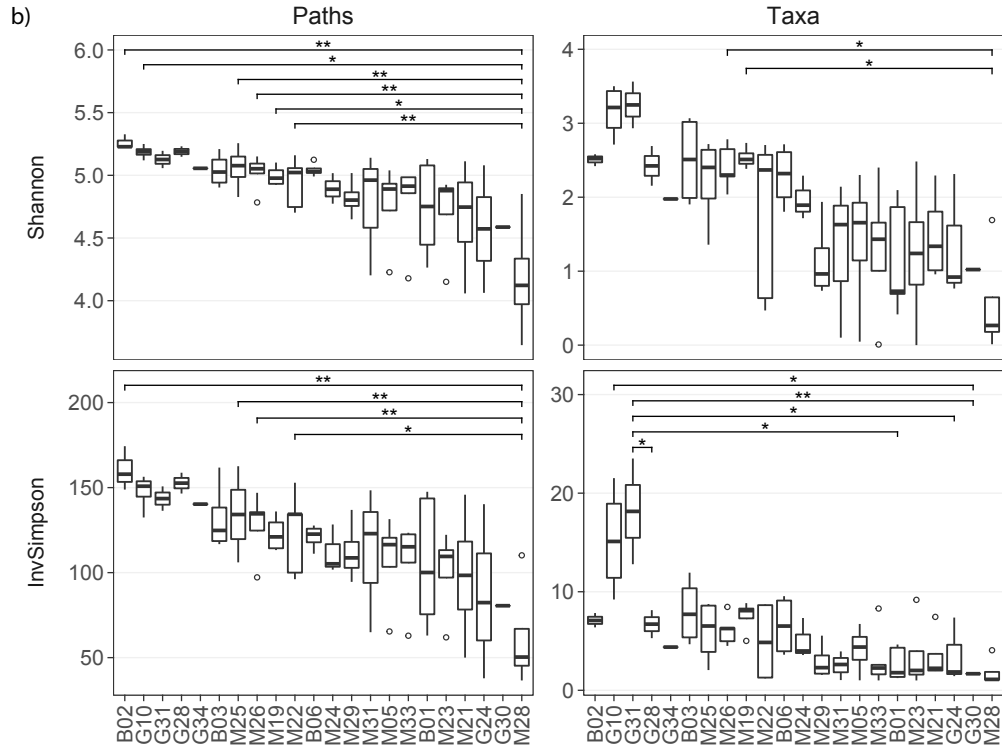

Supplement: Supplementary file 1 [file microorganisms-08-01003-s001.zip › Supplementary /Figure_S4.pdf]
